# Completely noninvasive multi-analyte monitoring system for cell culture processes

**DOI:** 10.1007/s10529-024-03521-z

**Published:** 2024-08-20

**Authors:** Vida Rahmatnejad, Michael Tolosa, Xudong Ge, Govind Rao

**Affiliations:** Center for Advanced Sensor Technology, Department of Chemical, Biochemical and Environmental Engineering, University of Maryland, Baltimore County, Baltimore, MD 21250 USA

**Keywords:** Dissolved carbon dioxide, Dissolved oxygen, Noninvasive, Sensor, Mammalian cell culture, Flow cell

## Abstract

Although online monitoring of dissolved O_2_, pH, and dissolved CO_2_ is critical in bioprocesses, nearly all existing technologies require some level of direct contact with the cell culture environment, posing risks of contamination. This study addresses the need for an accurate, and completely noninvasive technique for simultaneous measurement of these analytes. A “non-contact” technique for simultaneous monitoring of dissolved O_2,_ pH, and dissolved CO_2_ was developed. Instead of direct contact with the culture media, the measurements were made through permeable membranes via either a sampling port in the culture vessel wall or a flow cell. The efficacy of the “non-contact” technique was validated in *Escherichia coli* (*E.coli)*, Chinese hamster ovary (CHO) culture processes, and dynamic environments created by sparging gases in cell culture medium. The measurements obtained through the developed techniques were comparable to those obtained through control methods. The noninvasive monitoring system can offer accurate, and contamination-minimized monitoring of critical process parameters including dissolved O_2_, pH, and dissolved CO_2_. These advancements will enhance the control and optimization of cell culture processes, promising improved cell culture performance.

## Introduction

Cell culture is a common practice in academia and the pharmaceutical industry and is conducted for different purposes, such as investigating the physiology or biochemistry of cells, studying the effect of drugs or chemicals on cells, fabricating artificial tissues, and manufacturing biologics. Any changes in the environmental condition of cells can affect the cell function. For example, the pH level in the cell culture medium directly impacts the enzymatic activity and metabolism of cells (Klein et al. 2021). Normal cells achieve optimal growth within an alkaline pH range, while cancer cells tolerate a wider pH range, including acidic environments. Therefore, maintaining an optimal pH specific to the cell culture process is crucial. Dissolved gases also impact cellular physiology. For example, high partial pressure of CO_2_ reduces pH, which affects cell metabolism and alters protein properties. Similarly, low partial pressure of CO_2_ negatively affects cell growth. Furthermore, studies show that hypoxia (less than 10% O_2_) promotes stem cell differentiation (Günter et al 2018; Schulz and Münzel 2011; Blombach and Takors 2015). Considering the importance of O_2_, CO_2,_ and pH in cell behavior, these analytes must be monitored throughout the cell culture process. The data obtained from sensors not only provide a thorough understanding of the cell culture environment but can also be utilized to develop control systems for maintaining a desirable level of critical process parameters. Furthermore, to ensure compliance, the Food and Drug Administration (FDA) encourages the use of process analytical technologies (PATs) in the biopharmaceutical industry through guidance on “Innovative Pharmaceutical Development, Manufacturing, and Quality Assurance”(FDA 2004). This has led to the development of various sensors for cell culture processes (Abou-el-Enein et al. 2021; Klein et al. 2021; Lashkari 2017; Rao. 2020).

Electrochemical sensors offer robust and efficient performance and are the most commonly used sensors for monitoring DO and pH. However, their bulkiness makes them less appropriate for small-scale cell culture processes. On the other hand, optical sensors are small and ideal for low-volume cultures. Their minimally invasive nature reduces the chance of contamination. However, they still require direct contact with the cell culture environment to conduct the measurements. Single on-chip sensor and sensing cell culture flask (SCCF) sensor are newly developed techniques for monitoring pH and DO. In another novel technique, a Clark-type DO sensor is coupled with a BLE chipset (a microcontroller used for data processing and transmission) where the chip is embedded in the bottom of the vessel. Furthermore, Wavepod II-pHOPT from GE Healthcare, iTube pH Bioreactor from PreSens, TurFluor pH from Fitnesse, and OptiSens pH from Sartorius are among the commercially available optical sensors. An optical sensor consisting of a sensor cassette, a pump, and a flow-through cuvette was recently reported. In this technique, the sample is transferred to an LED cassette; therefore, no contact with the cell culture environment is required. The measurements via this method have high accuracy and sensitivity. However, errors could result from indicator-protein binding or turbidity from contamination (Papantoniou et al. 2018; Fuentes et al. 2022; Tanumihardja et al. 2021; Kieninger et al., 2018; Al-Ani et al., 2018; Miller 1966; Stine et al. 2020; Kattipparambil Rajan et al. 2016).

Electrochemical and optical sensors are commonly utilized as monitoring systems for monitoring DCO_2_ throughout the cell culture process. Off-gas analyzers offer an alternative technology for monitoring DCO_2_ without direct contact with the cell culture medium. This technique is inexpensive and highly stable; however, it does not provide real-time DCO_2_ values in the media. Another method for DCO_2_ monitoring is the circulation direct monitoring and sampling system (CDMSS). The technique allows sampling without interrupting culture agitation and can measure CO_2_ in gas and liquid phases. However, CDMSS requires a system to prevent bypass component clogging and is not appropriate for small volumes of cultures (Kroll et al. 2019; Takahashi et al. 2017).

Microfluidic systems are another type of technology developed for monitoring different analytes in bioreactors. In these techniques, the sample is transferred to the sensor for measurements. Some examples of this type of technology are biophotonic lab-on-a-chip for pH monitoring, multi-sensor microsystem for monitoring pH and DO, magnetic optical sensor particles (MOSePs) and Chip-based monitoring system designed for monitoring DO. Hydrogel microarray sensor has been reported for monitoring DO and pH via optical sensors positioned externally to the bioreactor. This technique offers reliable measurements; however, in this method, the sensing part comes in direct contact with the cell culture medium, which has the potential to impact the cell culture medium. Flow loop developed by SBI is a commercially available technology for monitoring DO and pH throughout the process. This method enables the monitoring of DO and pH from outside of the vessel and can be adjusted for various types of vessels. However, one drawback of this technology is that the luminescent dye is in direct contact with the cell culture medium throughout the process. This raises concern regarding the cytotoxicity of the dye. In general, all the aforementioned methods are limited to monitoring one or two critical analytes and require the integration of the sensing components with the cell culture vessel (Coluccio et al. 2019; Munoz-Berbel et al. 2013; Lee et al. 2008).

From the above discussions, it can be seen that nearly all existing technologies require some level of contact with the cell culture environment, posing a risk for contamination or interferences. The purpose of this study is to develop a completely noninvasive “non-contact” sensing technology that is capable of online monitoring of DO, pH, and DCO_2_ while addressing the limitations of the discussed technologies. The techniques for noninvasive measurement of DO and DCO_2_ were developed in the authors’ lab and reported in previous publications. For noninvasive monitoring of DO, a sensing patch is placed in a sealing part and attached outside the cell culture vessel. During the process, oxygen diffuses through the vessel wall and is detected by the optical sensing patch. The noninvasive technique for monitoring DCO_2_ works based on measuring the initial diffusion rate of CO_2_ through a silicone membrane in the wall of the cell culture vessel. In both techniques, the measurements are conducted without requiring direct contact with the cell culture medium. The sampling approach is cost-effective and compatible with various types of single-use vessels (Gupta et al. 2013; Rahmatnejad et al. 2022).

A novel technique for noninvasive monitoring of pH was developed by the authors and is discussed in this paper. The measurement is made by a pH sensing patch through a semi-permeable cellulose membrane. So, there is no direct contact between the pH-sensing patch and the cell culture media. Subsequently, a flow cell technology for monitoring DO, pH and DCO_2_ is described. The flow cell conducts online and simultaneous monitoring of these process parameters from outside of the cell culture vessel, and the measurements are conducted based on the individual noninvasive methods developed for each analyte.

The flow cell technology addresses the major challenges associated with existing monitoring systems. One major advantage of this technology is the elimination of contamination risks. This is specifically important in the manufacturing process of cell therapies, where maintaining a contamination-free process in compliance with GMP regulations is critical (Abou-el-Enein et al 2021; Barone et al. 2020). Furthermore, in this technique, the risk of cytotoxicity associated with sensing parts is minimized because measurements are conducted through membranes, and the fluorescent dye within the sensing patches does not contact the cell culture medium directly. The technique can be utilized in cultures with different working volumes, accommodating a wide range of processes using various types of bioreactors. Another advantage of this method is the ease of replacing the malfunctioning parts without interrupting the cell culture process.

## Methods and materials

### Analytics

#### Optical measurement system

Optical sensors, consisting of electronics and sensing patches, were utilized for measuring DO and pH. The pH sensing patch includes a fluorescent dye, 6,8-dihydroxypyrene-1,3-disulfonic acid disodium salt, immobilized in a hydrogel matrix (Ge et al. 2012). The excitation spectrum of the dye changes in response to variations in the pH of the solution (Vallejos et al. 2010). The technique for online measurement of pH is a ratio-metric method wherein the pH value of the media is correlated with the corrected ratio of the emission intensities at two excited wavelengths of 468 nm and 408 nm. In our previous study on low-cost calibration-free pH sensing (Ge et al. 2012), it was found that the brightness of the violet and blue LEDs used to build the detectors for excitation was not completely uniform, which could introduce inter-device differences. To solve this problem, we introduced an algorithm to correct the effect of LED brightness on the ratio of fluorescence intensities, referred to as the corrected ratio (Ge et al. 2012). For the DO patch, the sensing properties of the fluorophore, tris-(bathophenanthroline) ruthenium(II) chloride, are influenced by alterations in the DO concentration (Ge and Rao 2012; Tolosa et al. 2002). For evaluation of the noninvasive method developed by the authors, control and noninvasive measurements were conducted simultaneously. The DO and pH patches were autoclaved at 121 °C for 20 min before conducting measurements. For control measurements, the patches were attached inside the cell culture vessel (Ge and Rao 2012). For noninvasive measurements, the patches were attached to the samplers outside the cell culture vessel. The preparation of the samplers is described in later sections. To conduct measurements through control and noninvasive methods, readers were placed below the vessel, and the LED light was aligned with the sensing patches. During the process, in noninvasive techniques, oxygen and protons pass through the permeable membranes of silicone and cellulose, respectively. The LED light emitted by the readers is an excitation source. Upon excitation by the LED light, the dyes within the patch emit light which is detected, analyzed, and converted to the appropriate readings.

#### Rate-based measurement system

A rate-based technique was utilized for conducting online measurement of CO_2_ in the flow cell. This technique works based on correlating the CO_2_ concentration in the cell culture medium with the diffusion rate of the CO_2_ through the silicone membrane (Chatterjee et al. 2015). In this method, CO_2_ passes through the silicone membrane, is collected in the sampler, and is transferred to the sensor for measurements. The method was previously evaluated by Chatterjee et al. 2015 and Rahmatnejad et al. 2022, and the results indicate the effectiveness of the technique (Rahmatnejad 2021; Rahmatnejad et al. 2022; Chatterjee et al. 2015).

#### Sensor calibration

The pH sensor calibration was conducted by attaching a pH sensing patch to the bottom wall of the vessel and introducing buffers with pH values ranging from 5.5 to 8.5. The corrected ratio corresponding to each pH value was measured and recorded. The relationship between pH value and corrected ratio was determined through regression interpolation.

The CO_2_ sensor calibration was conducted by sparging different percentages of CO_2_ (0.0%, 2.5%, 5.0%, 7.5%, 10.0% for mammalian cultures and 0.0%, 5%, 10.0%, 15.0%, 20.0% for microbial fermentation) into the medium. For DO sensor calibration, a DO sensing patch was attached to the bottom wall of the vessel. Subsequently, different percentages of O_2_ were sparged into the medium by combining different percentages of air (0.0%, 20%, 40%, 60%, 80%, and 100%) and nitrogen. In both calibration processes, the gas mixtures were created using two mass flow controllers (Digital Pressure Controller, Single-Valve, 0–30 psia, Cole-Parmer, Vernon Hills, IL, USA).

The percentage of gases sparged into the medium was converted to the concentration of dissolved gases utilizing Henry’s Law relation. Henry’s law constants were obtained from the compilation of Henry’s law constants (Sander 2015). For each percentage of CO_2_ sparged, the initial diffusion rate of CO_2_ through the silicone membrane was measured using the LabVIEW software developed by the authors. Regression interpolation was then utilized to convert measurements into concentrations of the respective gases.

### Noninvasive monitoring of pH

#### T-flask setup preparation

**Fig. 1:**
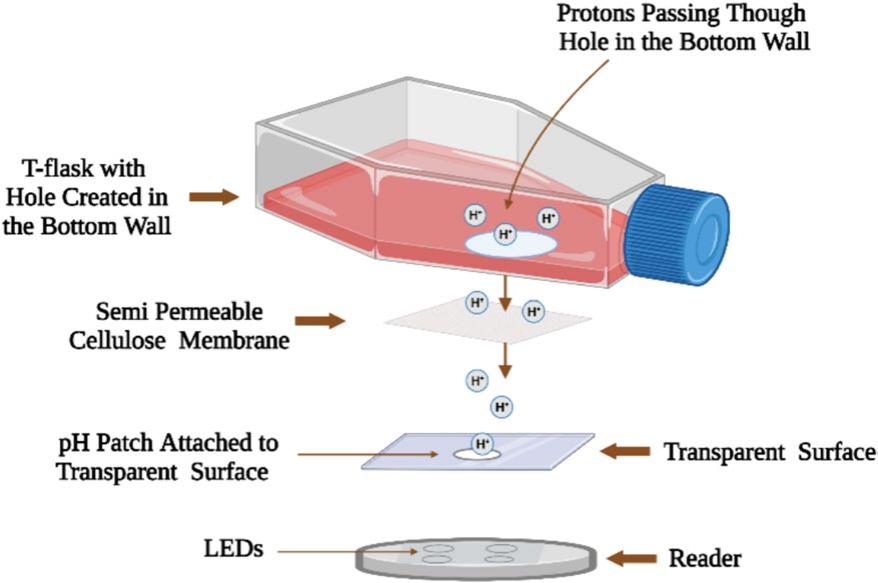
T-flask setup for noninvasive monitoring of pH. Throughout the process, protons diffuse through the cellulose membrane, contact the sensing patch, and measurements are conducted based on the optical sensing technique. Figure created with BioRender.com

To prepare the setup for noninvasive measurement of pH, a hole was created in the bottom wall of a T-flask. Subsequently, a cellulose membrane (Fisher Scientific, Hampton, NH, USA) was attached externally over the hole. A sampler, consisting of a pH sensing patch attached to a transparent layer, was attached to the cellulose membrane from outside. The sensing patch was aligned with the center of the hole. The semi-permeable membrane has a pore size of 4.8 nm and molecular weight cut off (MWCO) of 12,000 Daltons allowing the small-molecule components of the cell culture medium to move towards equilibrium concentration on both sides of the membrane. Different parts of the setup are shown in Fig. 1. To conduct the online measurement of pH, a reader was placed below the vessel. The LED light must be aligned with the pH patch in the sampler.

After preparing the setup, a pH patch was attached to the bottom wall of the modified T-flask, as a control method for pH measurement. Different buffers with varying pH values were added to the T-flask. After adding each solution, the measurements through control and noninvasive methods were recorded. The response times, representing the time taken for the sensor to reach 90% of the output, were calculated for both methods.

#### Long-term exposure of the cellulose membrane to the medium

To investigate the impact of the exposure of the cellulose membrane to the cell culture medium on the pH measurements, pre-calibration (calibration before exposure to the cell culture medium) and post-calibration (calibration after exposure to the cell culture medium) were conducted through noninvasive technique. After pre-calibration, 10 ml of complete medium comprised of 10% v/v Fetal Bovine Serum (FBS) (ATCC, Manassas, VA, USA) and 90% v/v of Dulbecco’s Modified Eagle’s Medium (DMEM) (ATCC, Manassas, VA, USA) was added to a modified T-25 flask. The T-flask was then placed in the 5% CO_2_ incubator and maintained for 10 days. On day 10, the medium was removed, and the T-flask was rinsed with deionized (DI) water before the post-calibration process was performed. The calibration processes were conducted based on the procedures described previously.

#### Cell attachment on cellulose membrane

The noninvasive pH measurements are conducted through a cellulose membrane. To study whether the pH measurements are affected by cell attachments in the culture process of adherent cells, cell attachment on the cellulose membrane was investigated by conducting DAPI staining on the membrane. For this purpose, 2 cm × 2 cm pieces of the cellulose membrane were placed in 6 wells of a 6-well-plate. In each well, adherent Chinese hamster ovary (CHO-K1) cells (ATCC, Manassas, VA, USA) were cultured in 3 ml of a complete medium composed of 10% v/v Fetal Bovine Serum (FBS) (ATCC, Manassas, VA, USA), and 90% v/v HAM’s F12 medium with L-Glutamine (Lonza, Walkersville, MD, USA). The seeding density was $3.1\times {10}^{4}$ cells/cm^2^, and one piece of membrane was harvested each day on days 3, 4, 5, 6, 7 and 8. Cells on membranes were fixed in 4% Paraformaldehyde (TissuePro Technology, Gainesville, FL, USA), and membranes were stored in Phosphate Buffered Saline (PBS) (Thermo Fisher Scientific, Waltham, MA, USA). For DAPI staining, a 300 nM DAPI solution was prepared by dissolving the content of the vial in 2 ml of DI water and subsequent dilution in PBS. The cellulose membranes were stained by adding 300 µl of the diluted DAPI solution, followed by 5 min incubation and rinsing with PBS three times. The stained membranes were then imaged using a fluorescence microscope.

#### Noninvasive pH measurement in CHO-K1 cell culture process

A modified T-flask prepared based on the process explained in Fig. 1, was used as cell culture vessel in this experiment. The T-flask is designed to measure pH in a noninvasive way. As a control method for pH measurement, a pH sensing patch was attached inside the modified T-flask. Subsequently, CHO-K1 cells were seeded in the T-flask with a working volume of 53 ml and seeding density of $2.85\times {10}^{4}$ cells/${cm}^{2}$. The cell culture process was conducted in a 5% CO_2_ incubator set at 37 °C, and pH was simultaneously monitored through both control and noninvasive methods.

#### Noninvasive pH measurement in *E. coli* culture process

Fifty μl of BL21(DE3) *E. coli* (Invitrogen, Waltham, MA, USA) was added to 50 ml of LB Lennox medium in a 200 ml shake flask. The medium contained 10 g tryptone, 5 g yeast extract, and 5 g sodium chloride per liter. The cells were grown at 37 °C and 180 rpm for 20–24 h. The setup described in Fig. 1 served as the cell culture vessel. For monitoring DO and pH through the control method, sensing patches were attached to the bottom wall of the T-flask. The pH measurements were simultaneously conducted through the noninvasive technique. In this experiment, the preculture was inoculated in the vessel, and an initial optical density (OD) of 0.65 in a working volume of 250 ml was achieved. The culture was conducted at an agitation speed of 200 rpm at 37 °C, and 25 μl of Kanamycin was added to the T-flask to isolate the *Escherichia coli* (*E. coli)* bacteria.

### Flow cell

#### Flow cell setup preparation

**Fig. 2:**
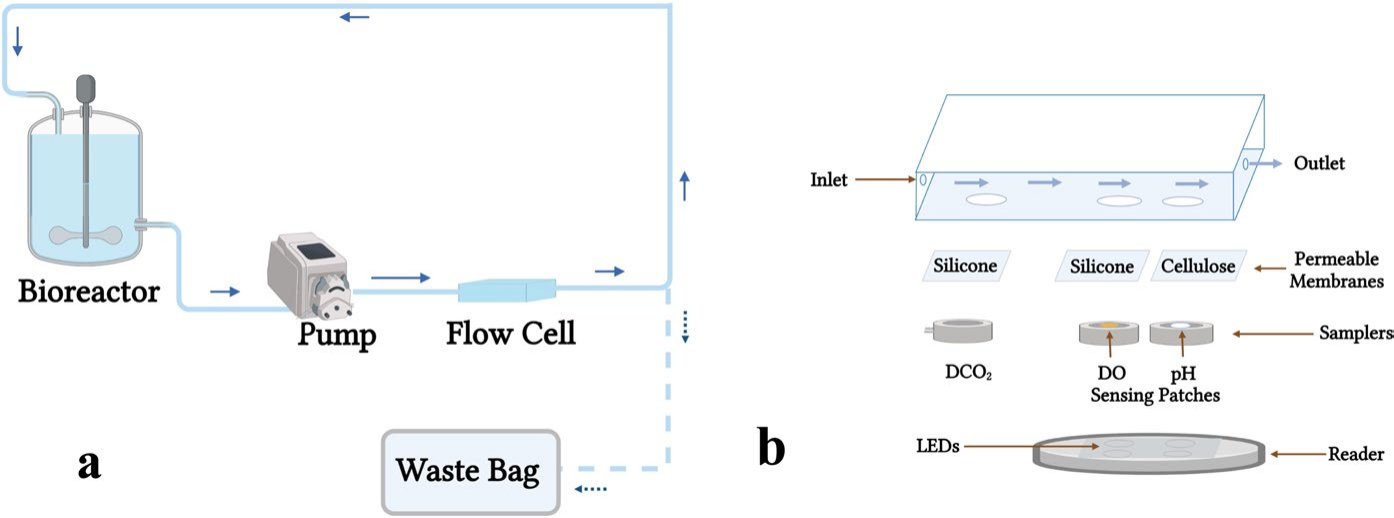
Flow cell setup. **a** The sample is drawn from the bioreactor and transferred to the flow cell to conduct simultaneous measurements of dissolved O_2_ (DO), pH, and dissolved CO_2_ (DCO_2_). **b** Different parts of the flow cell including membranes and samplers are shown in this figure. The DO, pH, and DCO_2_ measurements are conducted simultaneously as the sample passes through the flow cell. Figures created with BioRender.com

To conduct the simultaneous monitoring of DO, pH, and DCO_2_ from outside of the cell culture vessel, a flow cell technology was developed. The online measurements for different analytes are conducted as the sample passes through the flow cell. After the sample exits the flow cell, it is returned to the bioreactor or transferred to the waste bag. Figure 2a shows the flow cell setup. The flow cell features three holes in the bottom wall. Two silicone membranes permeable to O_2_ and CO_2_, and a cellulose membrane permeable to proton were attached to the holes externally. The flow cell and samplers were fabricated from acrylic sheets. The DCO_2_ sampler includes a cavity in the center for collecting the CO_2_ gas diffusing from the silicone membrane, and two channels, for transferring the gas to the sensor. DO and pH samplers are transparent layers with corresponding patches attached to them. All three samplers were externally attached to the membranes. To conduct online measurements, the flow cell was placed on the reader, and the DO and pH patches were aligned with the LED lights. A schematic of the flow cell is shown in Fig. 2b.

#### Flow cell measurements

The LB broth medium was prepared by suspending 20 g of LB broth powder (Thermo Fisher Scientific, Waltham, MA, USA) in 1 L purified water. Different percentages of O_2_ and CO_2_ were sparged in the LB broth medium. The medium was continuously recirculated between the T-175 flask and the flow cell utilizing a peristaltic pump. DO, pH, and DCO_2_ were simultaneously measured from inside the flask and through the flow cell. The dimensions of the flow cell utilized were 9 cm L × 3 cm W × 1 cm H, and the flow rate for the sample was 0.25 ml/s.

For evaluating the pH measurements through the flow cell, a pH sensing patch was attached inside the T-flask as a control method. 200 ml of LB broth medium was added to the T-flask, and different percentages of CO_2_ (0%, 10%, 20%, and 2.5%) were sparged into the medium. Online measurements through the flow cell and control method were simultaneously conducted while the medium was continuously recirculated between the flow cell and the cell culture vessel.

To evaluate the efficacy of the flow cell in measuring CO_2_, 700 ml of LB broth medium was added to a vertically positioned T-175 flask. Various percentages of CO_2_ (0%, 20%, 40%, 60%, 80%, and 100%) were sparged into the medium. The medium was continuously recirculated between the flow cell and the T-flask. Control measurements were obtained directly inside the vessel through the rate-based technique via a silicone sampling loop submerged in the cell culture medium, and simultaneous measurements were conducted through the flow cell.

For evaluating DO measurements through the flow cell, 200 ml of LB broth medium was added to the T-flask, and different percentages of O_2_ (20%, 15%, 10%, 5%, and 0%) were sparged into the medium. Flow cell and control measurements were simultaneously conducted while the medium was continuously recirculated between the flow cell and the cell culture vessel.

In all experiments, gas mixtures were created through two mass flow controllers (Digital Pressure Controller, Single-Valve, 0–30 psia, Cole-Parmer, Vernon Hills, IL, USA).

#### Flow cell measurements in *E. coli* culture process

DO and pH patches were attached to the bottom wall of the 2000 ml shake flask to conduct control measurements from inside the culture. Subsequently, *E. coli* was inoculated into the shake flask with a working volume of 1000 ml*.* The agitation speed and temperature were set at 180 rpm and 37 °C, respectively. The initial optical density (OD), measured at 600 nm, was 0.9. To measure DCO_2_ through the control method, a silicone sampling loop was submerged in the cell culture medium, and the online measurements were conducted through the rate-based technique. The sample was continuously recirculated between the flow cell and the shake flask with a flow rate of 0.25 ml/s.

#### Flow cell delay

The flow cell measurements are conducted by transferring the sample from the cell culture vessel to the flow cell outside the cell culture vessel. Therefore, a delay for flow cell measurements is expected. Different factors, such as the length of the transfer tube, flow rate, and volume of the flow cell, contribute to the delay in flow cell measurements. The time required for transferring the sample to the flow cell can be calculated using the relationship below:

1 $$Q = \frac{Ad}{t} \to t = \frac{Ad}{Q},$$

where:

Q: Flow rate.

A: Area of the cross-section of the tube.

d: Length of the tube.

t: Time.

The residence time of sample in the flow cell could be calculated by the relationship below:

2 $$R_{T} = \frac{V}{Q} ,$$

where:

$${R}_{T}$$: residence time.

V: Volume of the flow cell.

Q: flow rate.

According to Fick’s second Law, the following equation can be obtained which roughly estimate the time required for diffusion through membranes (Calculator Academy 2024):

3 $$\Delta t \approx \frac{{\Delta x^{2} }}{2D},$$

where:

$\Delta t$: Time for diffusion of the specific analyte (s).

$\Delta x$: Thickness of the membrane.

D: Diffusion coefficient.

## Results and discussion

### Noninvasive measurement of pH

The technique for noninvasive monitoring of pH was developed by placing a cellulose membrane between the cell culture medium and a pH-sensing patch. During the process, protons diffuse through the membrane and contact the sensing patch. The measurements are subsequently conducted based on the method described previously. The efficiency of the technique was studied by adding solutions with different pH values to the modified T-flask.

**Fig. 3:**
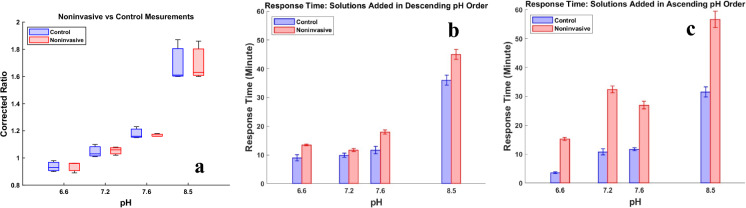
pH measurements through noninvasive technique (N = 4). **a** Comparison between measurement through control method and noninvasive method. The corrected ratio on the y-axis is the raw value of the pH sensor reading. **b** Response times in control and noninvasive methods when the solutions were added to the vessel in descending order of pH values. **c** Response times in control and noninvasive methods when the solutions were added to the vessel in ascending order of pH values. The boxes in 3a extend from the 25th to the 75th percentile of each group's distribution of values, horizontal lines in the boxes indicate the median values, and vertical extending lines indicate the most extreme values of each group. The error bars in 3b and 3c indicate the standard deviation of the corresponding data sets

Figure 3a demonstrates the measurements through the control and noninvasive methods. Figure 3b and c illustrate the response times for control and noninvasive methods when the solutions were added in descending order of pH values and ascending order of pH values, respectively. As it is shown in Fig. 3a, the measurements through noninvasive techniques are comparable with control measurements. The paired samples t-test was performed, and the calculated p-value of 0.9954 confirms that there is no significant difference between the control and noninvasive methods. Similarly, the p-values were obtained for response times in Fig. 3b and c by performing paired samples t-test and the results are respectively 0.4408 and 0.0014. These results indicate that there is significant difference between response times when solutions were added in ascending order which is due to the faster diffusion of protons into the patch compared to their outward diffusion. However, the results show no significant difference between the two methods when the solutions were added in descending order.

### Long-term exposure of the cellulose membrane to the medium

**Fig. 4:**
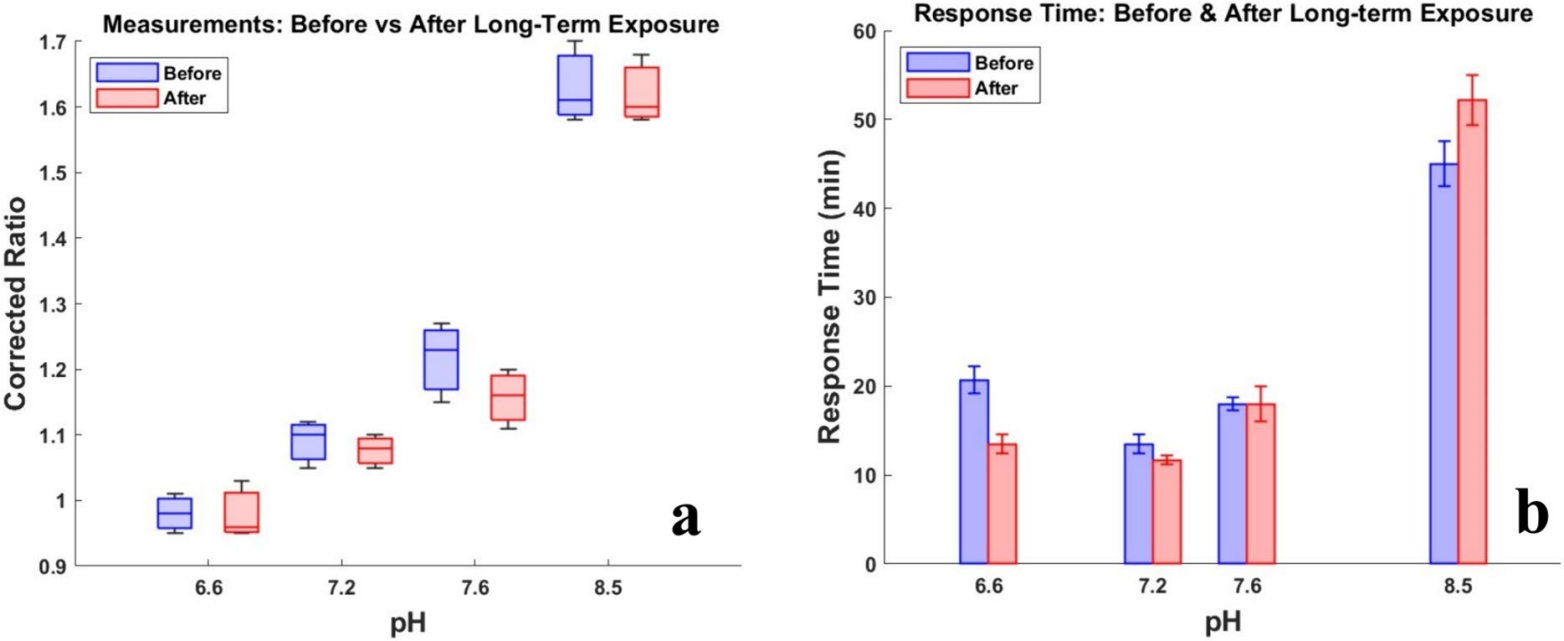
Noninvasive pH measurements before and after long-term exposure of the membrane to the cell culture medium (N = 4). **a** Comparison of measurements conducted via noninvasive method before and after the exposure of the cellulose membrane to the cell culture medium. The boxes in this figure extend from the 25th to the 75th percentile of each group's distribution of values, horizontal lines in the boxes indicate the median values, and vertical extending lines indicate the most extreme values of each group. **b** Calculated response times for the noninvasive method before and after exposure of the cellulose membrane to the cell culture medium. The error bars in this figure indicate the standard deviation of the corresponding data sets

In the noninvasive method for monitoring pH, the cellulose membrane is in direct contact with the cell culture medium. A study was conducted to investigate the effect of long-term exposure of cellulose membrane to the cell culture medium on the pH measurements and the response time. Figure 4a and b, respectively, show the measurements and response times before and after exposure to the cell culture medium. The p-values obtained by performing paired t-test for data shown in Fig. 4a and Figure b are 0.8583 and 0.8589, respectively. This indicates that there is no significant difference between the noninvasive measurements before and after the long-term exposure to the membrane. Therefore, the exposure of the membrane to the medium does not affect the measurements through the noninvasive method.

### Cell attachment on cellulose membrane

**Fig. 5:**
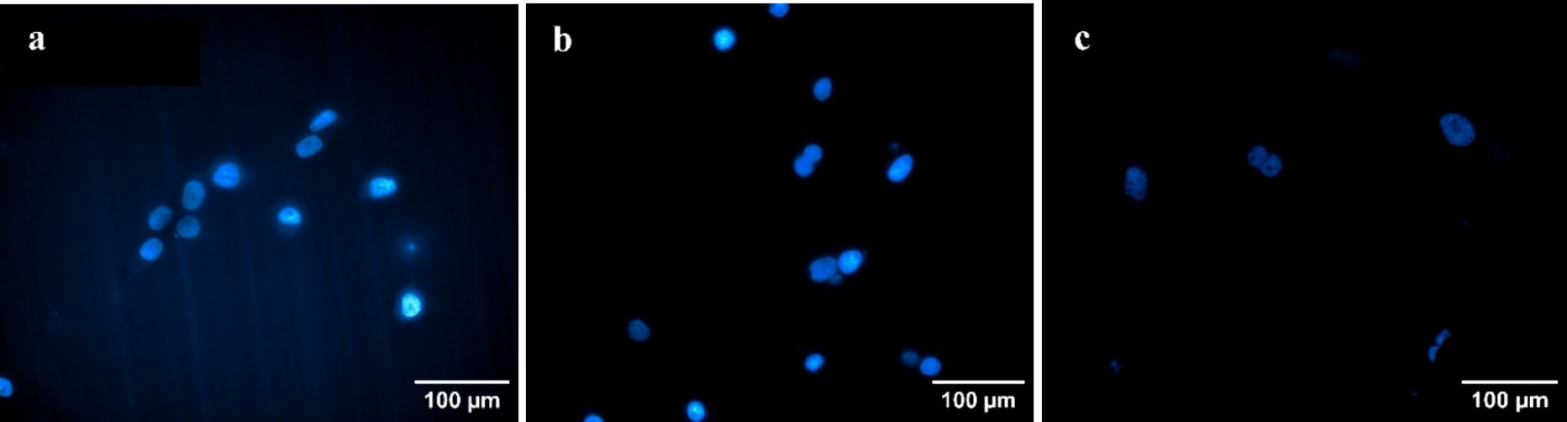
CHO-K1 Cell attachment on the cellulose membrane. Cell attachment on days **a** 6, **b** 7, and **c** 8 of the culture process was negligible. No cell attachment was observed in the first 5 days of the culture

In the noninvasive technique for measuring pH through cellulose membrane, the attachment of cells on the membrane was studied to investigate whether the measurements are affected by cell attachment when culturing adherent cells. Results from DAPI staining indicate no cell attachment on cellulose membranes on days 3, 4, and 5 of the culture process. On days 6, 7, and 8, only negligible cell attachment was found and shown in Fig. 5a, b, c, respectively. These results indicate the effectiveness of the noninvasive technique for pH measurement in the culture process of adherent cells.

### Noninvasive pH measurement in CHO-K1 cell culture process

**Fig. 6:**
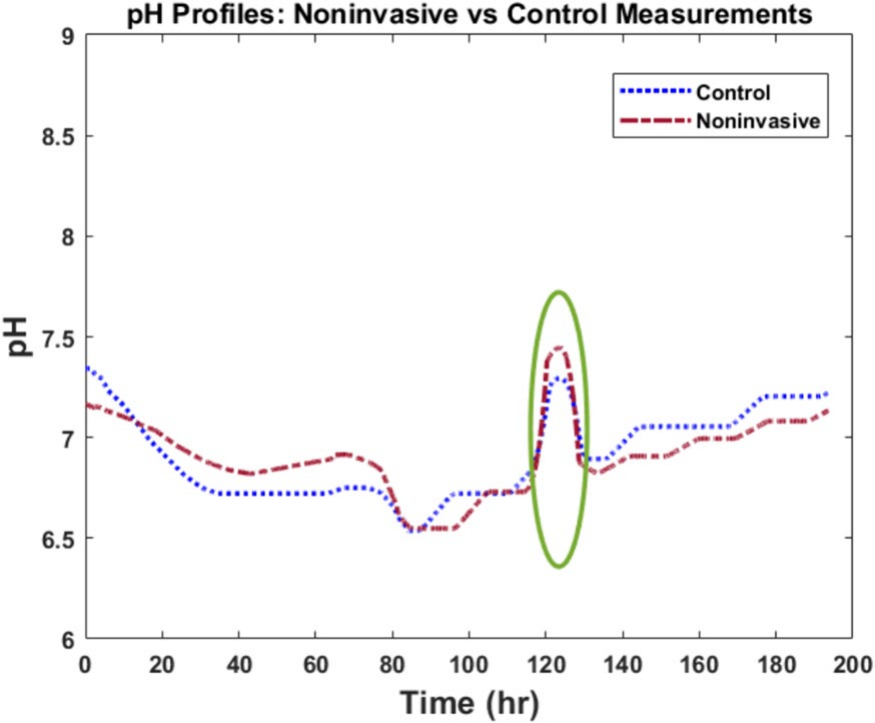
pH profiles obtained from CHO-K1 culture process. Comparison of the pH profile obtained from the noninvasive method (red) and control method (blue) throughout the CHO-K1 culture process

During 7 days of the CHO culture, pH was monitored through control and noninvasive methods simultaneously. The pH profiles obtained from both techniques are shown in Fig. 6.

Seeding density was $2.85\times {10}^{4}$ cells/${cm}^{2}$ and final density reached $7.1\times {10}^{4}$ cells/${cm}^{2}$ indicating cell growth. Both pH profiles in Fig. 6 show a decrease in pH during the first part of the culture until time point of 85 h. This is potentially due to the lactate and CO_2_ production during cell metabolism. An interesting event during the second part of the process, marked in Fig. 6, was the unintentional disconnection of the CO_2_ supply to the incubator in the time range of 115 h to 123 h. This led to a decline in the CO_2_ level inside the incubator which resulted in a decrease in the dissolved CO_2_ level and an increase in the pH level in the cell culture medium. After the CO_2_ supply was reconnected to the incubator, a gradual decrease in pH level was observed in the time range of 123 h to 133 h. This observation highlights the influence of CO_2_ on the pH in the cell culture medium and the efficacy of the noninvasive method in tracking pH changes in the cell culture medium (Shuler et al. 2017; Michl et al. 2019; Klein et al. 2022; Rogatzki et al. 2015; Naciri et al. 2008). Furthermore, the Pearson correlation between the noninvasive and control pH measurements is 90% which confirms the efficacy of the noninvasive technique.

### Noninvasive pH measurement in *E. coli* culture process

**Fig. 7:**
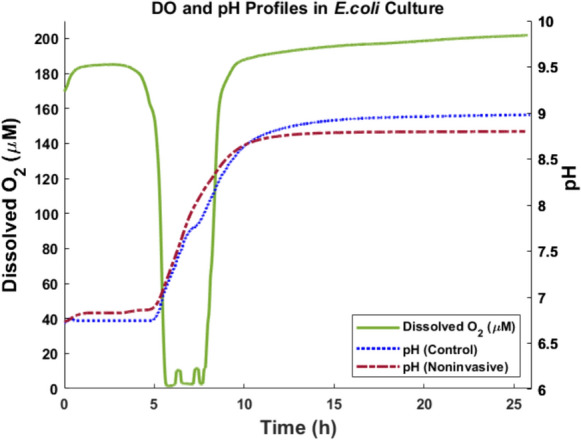
pH profiles obtained from *E.coli* culture process. The pH measurements were obtained through noninvasive (red) and control (blue) methods throughout the *E.coli* culture process. Dissolved oxygen was also monitored during the process (green). The results show the effectiveness of the noninvasive technique for pH measurement

The *E.coli* culture process started with an initial OD of 0.9 and reached 6.12 after 25 h, indicating cell growth. Figure 7 shows the pH profiles obtained through noninvasive and control methods.

In the initial phase of the culture, minimal change in pH profile is observed, which could be due to low cell metabolism. In the second part of the culture, between 5 to 10 h of the culture, an increase in pH profiles is observed, which is concomitant with a decrease in DO profile. This is potentially due to the cell growth and production of alkaline products (Ng 2018). When a protein-rich complex media is used, the cells cleave off ammonia from the contained amino acids as they have a much greater demand for the carbon. As a result, ammonium forms in the aqueous solution and causes an increase in pH. After the time point of 10 h, pH and DO profiles change in a smaller range. The Pearson correlation between the pH measurements from noninvasive and control methods is 98%, and this indicates that the noninvasive measurements are comparable with control measurements.

### Flow cell measurements

#### Sensor evaluations with medium

**Fig. 8:**
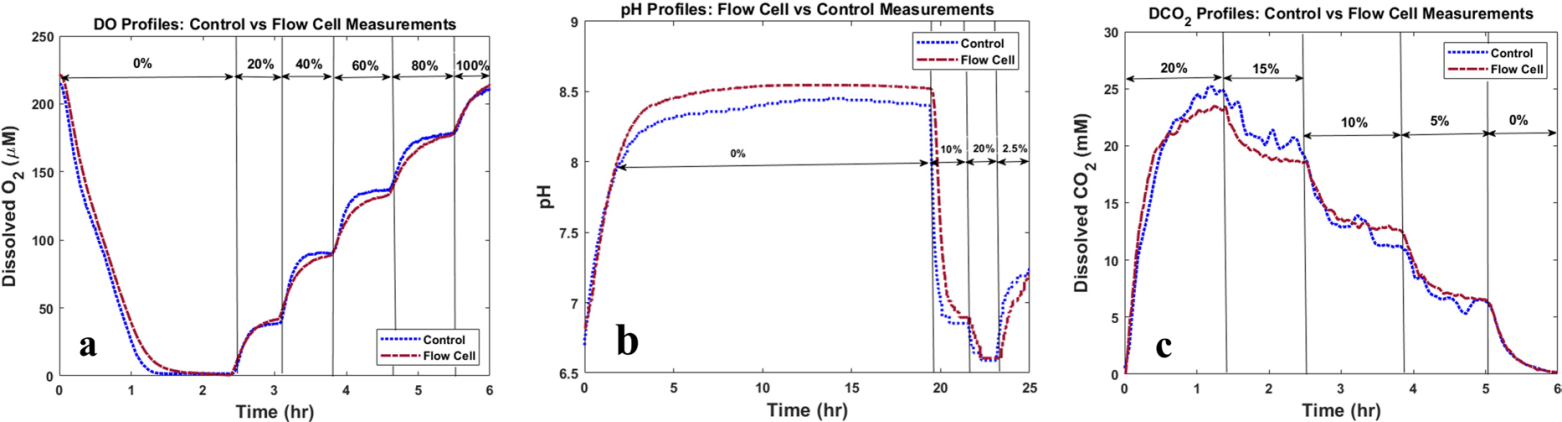
Dissolved O_2_ (DO), pH, and Dissolved CO_2_ (DCO_2_) measurements obtained through flow cell. **a** Comparison of Dissolved O_2_ (DO) profiles obtained from flow cell and control method. **b** pH profiles obtained from flow cell and control method. **c** Dissolved CO_2_ (DCO_2_) profiles obtained from the flow cell and via control method from inside the bioreactor

The online measurements of DO, pH, and DCO_2_ were obtained through flow cell and compared with control measurements. The measurements from both techniques are presented in Fig. 8a, b, c, respectively. The percentages of gases sparged in the cell culture medium are shown for different periods of time in each figure.

Changing the percentage of the gases sparged in the medium results in changes in the concentration of gases dissolved in the medium and the pH of the medium. The figures show that the profiles obtained through the flow cell and the control profiles are comparable. Furthermore, the Pearson correlation between control and flow cell measurements for DO, pH, and DCO_2_ are 99.57%, 98.27%, and 99.12%, respectively. This indicates that the flow cell is successful in tracking changes inside the cell culture vessel.

#### *E. coli* culture process

**Fig. 9:**
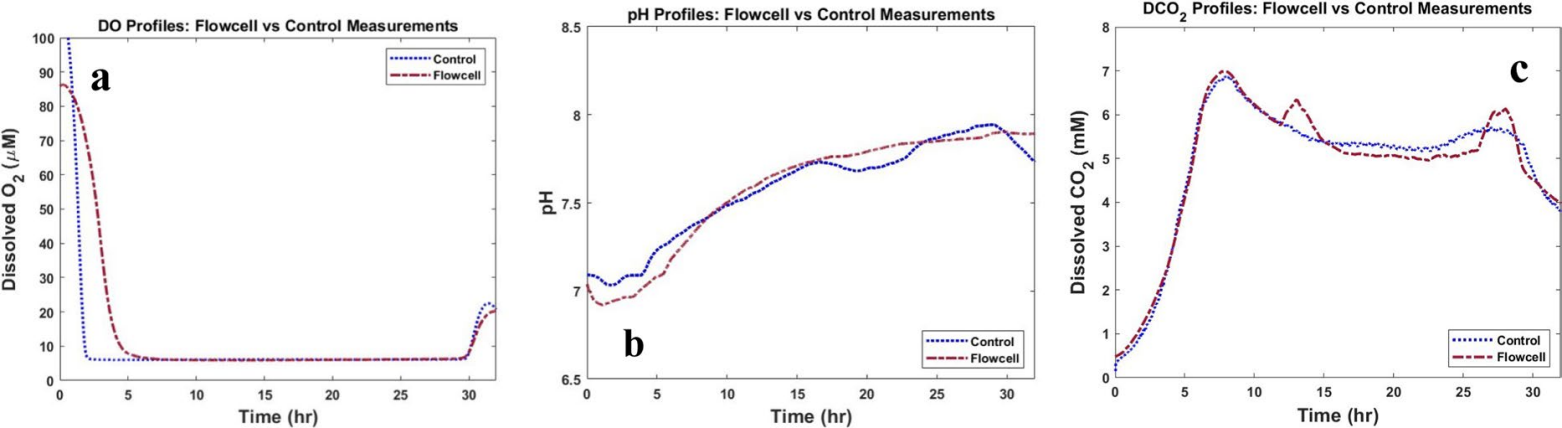
Flow cell measurements in *E. coli* culture process. During the *E.coli* culture process, medium including cells were recirculated between the shake flask and measurement of **a** Dissolved O_2_ (DO), **b** pH, and **c** Dissolved CO_2_ (DCO_2_) was conducted through flow cell and control method. Figures show that the profiles obtained from the flow cell are comparable with control measurements

*E. coli* was cultured in a 2L shake flask, and the medium was continuously circulated between the shake flask and the flow cell. During the process, DO, pH, and DCO_2_ were simultaneously measured through the control method inside the shake flask and the flow cell. Figure 9a, b, c depict the DO, pH, and DCO_2_ profiles. The Pearson correlations between control and flow cell measurements for DO, pH, and DCO_2_ are 61%, 73%, and 99%, respectively, confirming the efficacy of the flow cell in tracking changes inside the cell culture vessel.

In Fig. 9a, the initial delay observed for DO profile is potentially as a result of the formation of an air pocket within the DO sampler during the manufacturing process of the flow cell. This explanation seems true because the Pearson correlation obtained from the gas-sparge experiment described above using a different flow cell is very high (99.57%). Further research is necessary to pinpoint the cause of the delay and to enhance the measurement accuracy using the flow cell.

#### Flow cell delay

Throughout the *E.coli* culture experiment described in the Methods and Materials section, the flow rate for transferring the sample to the flow cell was 0.25 ml/s, the inner diameter of the transfer tube was approximately 0.31 cm, and the length of the tube transferring the sample to the flow cell was approximately 183 cm. Therefore, the time required for transferring the sample to the flow cell is approximately 58s calculated using Eq. (1). By considering 27 ml as the total volume of the flow cell, the residence time is approximately 108s estimated using Eq. (2). The thickness of silicone and cellulose membranes utilized in the flow cell was respectively 100 µm and 30 µm. Therefore, based on Eq. (3), the approximate time for diffusion of protons through cellulose membrane, diffusion of oxygen through silicone membrane, and diffusion of carbon dioxide through silicone membrane are respectively 16.7s, 1.5s, and 2.3s. The diffusion coefficient of 2.7×10^−7^ cm^2^/s for protons through cellulose membrane, 3.25×10^−5^ cm^2^/s for oxygen in silicone membrane, and diffusion coefficient of 2.2×10^−5^ cm^2^/s for carbon dioxide in silicone were obtained from literature and utilized in calculations (Fan et al. 2017; Markov et al. 2014; Yang and Kao. 2010).

## Conclusions

Although online monitoring of dissolved O_2_, pH, and dissolved CO_2_ is critical in bioprocesses, nearly all existing technologies require some level of direct contact with the cell culture environment, posing a risk of contamination. The “non-contact” monitoring system reported in this manuscript enables online monitoring of DO, pH, and DCO_2_ and can provide accurate results comparable to traditional methods. As there is no direct contact with the cell culture medium, it eliminates the risk of contamination. This feature is specifically crucial in cell therapy manufacturing processes, where the cells cannot be sterilized in the final stage. It also addresses the concern regarding the cytotoxicity of sensing patches, which are directly placed in the media in traditional methods. The design of the setup permits the replacement of malfunctioning parts of the monitoring system without interrupting the cell culture process. This makes it an appropriate monitoring system for long-term processes. Unlike currently available sensors, the application of the flow cell is not limited to specific cell culture processes, and has the potential to be used in different cell culture processes with different volumes (Papantoniou et al. 2018; Li et al. 2021; Esmonde-White et al. 2017; Reyes et al. 2022). Further studies are needed to optimize the design of the flow cell to reduce the residence time in the flow cell.
